# Emergence of Tigecycline Nonsusceptible and IMP-4 Carbapenemase-Producing K2-ST65 Hypervirulent Klebsiella pneumoniae in China

**DOI:** 10.1128/Spectrum.01305-21

**Published:** 2021-10-27

**Authors:** Yawei Zhang, Xiaojuan Wang, Qi Wang, Hongbin Chen, Henan Li, Shuyi Wang, Ruobing Wang, Hui Wang

**Affiliations:** a Department of Clinical Laboratory, Peking University People’s Hospital, Beijing, China; Johns Hopkins University School of Medicine

**Keywords:** tigecycline nonsusceptibility, carbapenem-resistant hypervirulent *Klebsiella pneumoniae*, IMP carbapenemase, fitness

## Abstract

Carbapenem-resistant hypervirulent Klebsiella pneumoniae (CR-hvKP) poses a significant public health challenge worldwide, but research on IMP-producing CR-hvKP and its tigecycline resistance is extremely scarce. We report herein the recovery of two IMP-4–producing, capsular serotype K2, sequence type 65 (K2-ST65), hypervirulent K. pneumoniae isolates (C1672 and C2051), which caused severe and fatal infections in ICU patients, after retrospectively screening 3,285 carbapenem-resistant K. pneumoniae isolates from 26 provinces in China. Notably, C2051 also demonstrated tigecycline nonsusceptibility, mediated by a frameshift mutation in the TetR/AcrR family transcriptional regulator. Both strains harbored *bla*_IMP-4_ and critical plasmid-borne virulence genes (*rmpA*/*rmpA2*, *iucA*, and *iroN*) and demonstrated high virulence in Galleria mellonella, indicating CR-hvKP. The *bla*_IMP-4_ gene was located on the IncU- and IncN-type plasmids, which showed high stability in C1672 and C2051 after serial passage for 5 days, with retention rates of 87% and 93.7%, respectively. No significant differences in growth rates were observed among the parental strains and the corresponding resistance plasmid-cured mutants (*P = *0.5273), suggesting that strains carrying the *bla*_IMP_ and virulence plasmids have the potential to exist for a long time without compromising fitness. The genetic environments of the *bla*_IMP-4_ gene in both strains were similar, and it has been inferred that the genetic regions of *bla*_IMP-4_ were inserted into different backbones. Several conjugal transfer genes, such as *traO*, *traE*, *traN*, and *traBCD*, were identified in the *bla*_IMP-4_-bearing plasmid of C2051, suggesting a higher ability for plasmid transmission. The convergence of IMP carbapenemase and tigecycline nonsusceptibility in a classic hypervirulent K. pneumoniae lineage highlights the need to enhance clinical awareness and epidemiologic surveillance.

**IMPORTANCE** To date, research on IMP-producing CR-hvKP is extremely scarce. Only one case of urinary tract infection caused by an IMP-6–producing K1-ST23 hypervirulent K. pneumoniae isolate in Japan was recorded, with a limited description of clinical information and genomic features. None of the published studies examined the virulence of the reported strains or the stability and fitness of resistance plasmids or presented a phylogenetic analysis. This dearth of data is notable because CR-hvKP infections are increasingly identified, but critical characteristics of the emerging resistance mediated by IMP carbapenemases in CR-hvKP remain unknown. Here, we report the emergence of two IMP-4 carbapenemase-producing K2-ST65 hypervirulent K. pneumoniae isolates that caused severe and fatal infections in clinical settings in China. Notably, one of them also demonstrated tigecycline nonsusceptibility. These strains carrying *bla*_IMP_ and virulence plasmids had the potential to exist for a long time without compromising their fitness, highlighting the urgent need to enhance clinical awareness and epidemiologic surveillance to prevent their dissemination.

## INTRODUCTION

The emergence of carbapenem resistance in hypervirulent Klebsiella pneumoniae strains poses a significant public health challenge worldwide (1). Carbapenem-resistant hypervirulent K. pneumoniae (CR-hvKP) is recognized as an important pathogen of clinical concern, with a propensity to cause severe and life-threatening infections (2). Although CR-hvKP cases have mainly been sporadically reported, a drift toward increasing prevalence has been observed (3), and such strains have disseminated rapidly in China (2–6).

Previous studies have elucidated that the CR-hvKP strains have evolved either from classic hypervirulent K. pneumoniae (e.g., capsular serotype K1, sequence type 23 [K1-ST23] and K2-ST65 clones) by acquiring resistance plasmids harboring carbapenemase genes or from carbapenem-resistant K. pneumoniae (e.g., K47-ST11 and K64-ST11 clones) by acquiring a pLVPK-like virulence plasmid (7). Furthermore, CR-hvKP has also emerged by acquiring a hybrid plasmid coharboring carbapenem resistance and virulence genes (8, 9). The most dominant carbapenem resistance genes detected in CR-hvKP are *bla*_KPC_ and *bla*_NDM_ (7). To date, research on IMP-producing CR-hvKP has been extremely scarce, with only one case recorded (10). Recently, Harada et al. described one case of urinary tract infection caused by an IMP-6–producing K1-ST23 hypervirulent K. pneumoniae isolate in Japan, underlining the importance of tracking such emerging resistance (10).

Carbapenem-resistant *Enterobacteriaceae* are often resistant to almost all available antibiotics, except tigecycline, colistin, and ceftazidime-avibactam (11). However, the clinical use of colistin is limited by toxicity and ceftazidime-avibactam is compromised by metallo-β-lactamase–producing bacteria, leaving tigecycline as the last therapeutic option (12). Studies on tigecycline resistance among CR-hvKP have been extremely limited until now. We searched the PubMed database using the terms “carbapenem-resistant hypervirulent K. pneumoniae” OR “carbapenemase-producing hypervirulent K. pneumoniae” AND “tigecycline” for articles published up to 1 June 2021 with no language restrictions. Only nine papers were found, four of which were on tigecycline nonsusceptible CR-hvKP, with the majority being identified in ST11 CR-hvKP strains (13–15). Here, we report the carriage of the carbapenemase gene *bla*_IMP-4_ in two K2-ST65 hypervirulent K. pneumoniae isolates in China, and notably, one of them demonstrated reduced tigecycline susceptibility mediated by an efflux pump. Importantly, to the best of our knowledge, this is the first known report of the emergence of tigecycline nonsusceptibility in CR-hvKP of the K2-ST65 lineage, highlighting the need for further surveillance.

## RESULTS AND DISCUSSION

### Clinical and microbiological characterization of strains C1672 and C2051.

Strain C2051 was hospital-acquired and recovered from a blood sample of a 74-year-old male patient with a history of appendectomy, pulmonary disease, and malignant lymphoma. The patient developed a liver abscess, endophthalmia, severe abdominal infection, intestinal obstruction, intestinal fistula, thrombocytopenia, bloodstream infection, and septic shock during hospitalization. He received treatment with imipenem plus central venous catheter, urinary catheter, mechanical ventilation, and gastric intubation; however, he finally died of multiple organ failure. A second strain, C1672, was recovered from the sputum of a 52-year-old man with ventilator-associated pneumonia in the intensive care unit. Combination antimicrobial therapy, including cefotaxime-sulbactam, moxifloxacin, and amikacin, was used for the treatment. The central venous catheter, urinary catheter, and tracheal cannula were inserted during hospitalization.

**FIG 1:**
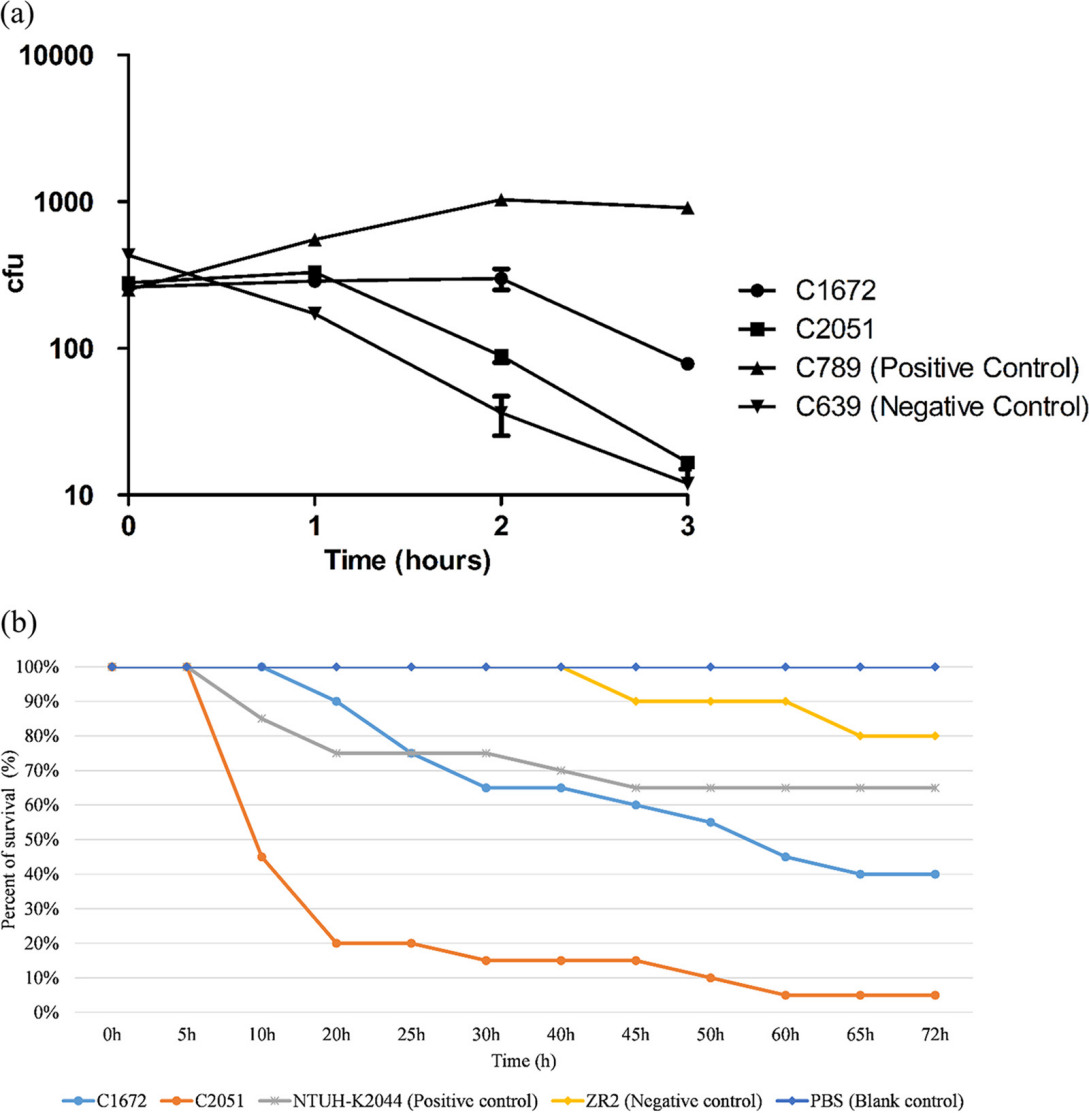
*In vitro* and *in vivo* virulence of C1672 and C2051. (a) Serum killing assay of C1672 and C2051. Strains from our previous study, C639 and C789, which were sensitive or resistant, respectively, to the serum killing, were used as negative and positive controls. Data are mean values ± standard errors of the means (SEM). (b) Virulence of C1672 and C2051 in the G. mellonella infection model. PBS, strain ZR2, and strain NTUH-K2044 were used as the blank, negative, and positive controls, respectively.

Both strains belonged to the K2-ST65 lineage and were resistant to third-generation cephalosporins and carbapenems due to their IMP-4 carbapenemase production. They were also intrinsically resistant to cefotaxime-avibactam. In addition, C2051 was not susceptible to tigecycline (MIC = 4 mg/liter). C1672 and C2051 presented as mucoid colonies on agar plates but tested negative for the string test, as the viscous strings formed were below 5 mm when stretching colonies using an inoculation loop. In addition, both strains exhibited intermediate resistance to serum killing *in vitro* (Fig. 1a). In the Galleria mellonella infection model, C1672 and C2051 demonstrated virulence higher than or similar to that of the positive-control strain NTUH-K2044 after 72 h of infection (Fig. 1b), suggesting that these isolates were hypervirulent. The patient demographics, clinical presentations, outcomes, and the microbiological features of these two strains are summarized in Tables 1 and 2.

**TABLE 1:** Clinical characteristics of two patients infected with IMP-4–producing CR-hvKP isolates in China

Characteristic	Value or information for patient from whom indicated isolate was obtained	
	C1672	C2051
Age (yr)	52	74
Sex	Male	Male
City	Xuzhou	Lanzhou
Hospital ward	ICU	ICU
Type of specimen	Sputum	Blood
Date of isolation	17 April 2016	23 December 2015
Clinical characteristic		
Underlying disease(s)	Unknown	Appendectomy, pulmonary disease, malignant lymphoma
Clinical presentation(s)	Ventilator-associated pneumonia	Liver abscess, endophthalmia, severe abdominal infection, intestinal obstruction, intestinal fistula, thrombocytopenia, bloodstream infection, septic shock
Laboratory findings		
Temp (°C)	38.3	39.1
White blood cell count/mm^3^	11.9 × 10^9^	10.88 × 10^9^
Indwelling devices	Central venous catheter, urinary catheter, tracheal cannula	Central venous catheter, urinary catheter, mechanical ventilation, and gastric tube
Outcome	Discharged	Died

**TABLE 2:** Microbiological characteristics of IMP-4–producing CR-hvKP isolates from two patients in China

Characteristic
	C1672	C2051
MIC (mg/liter) of:		
Cefoxitin	>256	>256
Cefotaxime	32	256
Ceftriaxone	128	256
Ceftazidime	256	>256
Cefepime	16	64
Piperacillin-tazobactam	4	256
Cefoperazone-sulbactam	64	128
Imipenem	1	2
Meropenem	2	2
Ertapenem	4	8
Amikacin	8	1
Ciprofloxacin	0.5	16
Levofloxacin	0.5	8
Minocycline	2	128
Chloramphenicol	>128	64
Fosfomycin	128	64
Aztreonam	256	256
Tigecycline	0.5	4
Colistin	0.25	0.5
Ceftazidime-avibactam^*a*^	>256	>256
Serotype and sequence type	K2-ST65	K2-ST65
String test	Negative	Negative
Resistance replicon; size (bp)	IncU; 328,921	IncN; 54,214
Resistance determinant	*bla*_IMP-4_	*bla*_IMP-4_
Virulence replicon; size (bp)	IncHI1B/IncFIB; 240,271	IncHI1B/IncFIB; 236,472
Major virulence genes		
Capsule regulators	*rmpA*, *rmpA2*	*rmpA*, *rmpA2*
Siderophores	*iroN*, *iutA*, *iucABCD*	*iroN*, *iutA*, *iucABCD*
Serum killing	Intermediate	Intermediate
Virulence in G. mellonella model	Similar to NTUH-K2044	Higher than NTUH-K2044

a Intrinsic resistance.

### The AcrAB-TolC efflux pump in tigecycline nonsusceptibility.

In the absence of tigecycline, the bacterial growth rates of strain C2051 with or without phenyl-arginine-β-naphthylamide (PAβN) were similar, suggesting that the presence of PAβN at a concentration of 25 mg/liter did not affect the growth of the bacteria. The addition of the efflux pump inhibitor PAβN reversed tigecycline nonsusceptibility, with a decrease in the MIC of tigecycline from 4 mg/liter to ≤0.5 mg/liter. PAβN is known to inhibit resistance-nodulation-division (RND) efflux pumps (16). Therefore, the nonsusceptibility to tigecycline in strain C2051 was likely related to the activity of efflux pumps.

The most-critical and best-studied efflux pump in members of the *Enterobacteriaceae* is AcrAB-TolC, which belongs to the RND family (17). It consists of the inner-membrane transport protein AcrB, outer-membrane channel TolC, and membrane fusion protein AcrA (17). The expression of AcrAB-TolC is mediated by a few local and global regulators, such as AcrR, MarAR, and SoxSR. Thus, we checked the mutations, insertions, or deletions in genes that mediate tigecycline resistance, namely, *acrA*, *acrB*, *acrR*, *marA*, *oqxAB*, *oqxR*, *lon*, *ramA*, *ramR*, *rarA*, and *soxS*. A frameshift mutation (2-bp deletion) in the TetR/AcrR family transcriptional regulator was identified in strain C2051. Furthermore, strain C2051 was negative for the plasmid-mediated tigecycline resistance mechanism *tet(X)*, which encodes the tetracycline-inactivating enzyme, and the RND-type efflux pump gene cluster *tmexCD1-toprJ1*, which was recently reported to confer resistance to tigecycline (18, 19). Meanwhile, none of the novel mobile tigecycline resistance mechanisms were discovered, as tigecycline-nonsusceptible transconjugants were not acquired in the conjugation assays.

### Stability of carbapenem resistance and its impact on fitness.

**FIG 2:**
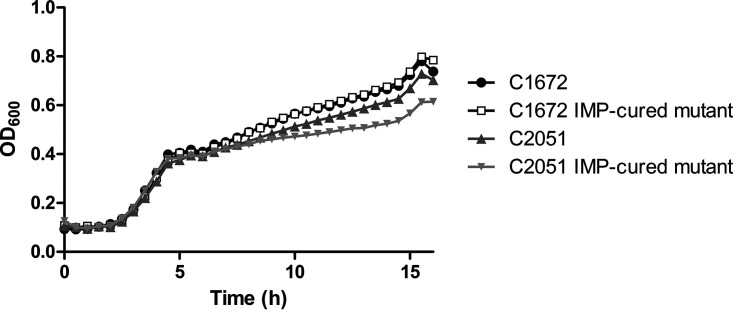
Fitness of C1672/C2051 and their corresponding plasmid-cured mutants. There were no significant differences in growth between the parental strains and the corresponding resistance plasmid-cured mutants (*P = *0.5273).

Infections caused by IMP-producing CR-hvKP have rarely been reported (10). We speculated that the cooccurrence of a *bla*_IMP_-bearing plasmid and a virulence plasmid may bring a high fitness cost to the host strain; therefore, resistance plasmid stability and strain fitness after acquiring resistance plasmids were evaluated. However, to our surprise, the resistance plasmid harboring *bla*_IMP_ demonstrated high stability after serial passage for 5 days in C1672 and C2051, with retention rates of 87% and 93.7%, respectively. Furthermore, no significant differences in the growth rates were observed between the parental strain and the corresponding resistance plasmid-cured mutants (*P = *0.5273) (Fig. 2). Taken together, these results indicate that strains carrying the *bla*_IMP_ gene and virulence plasmid have the potential to exist for a long time without compromising fitness, raising our concern about this emerging resistance in clinical settings.

### Genomic features.

**FIG 3:**
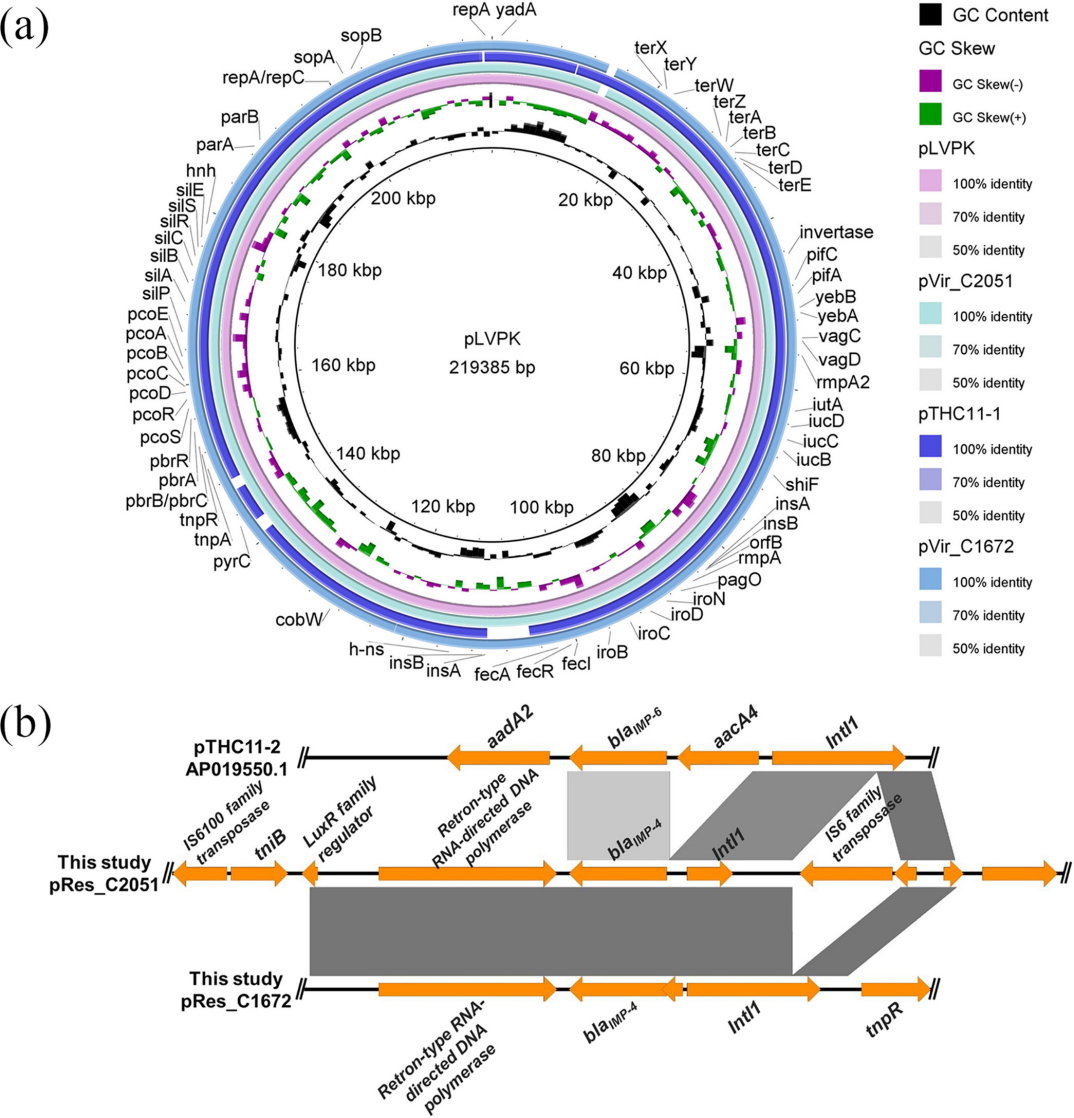

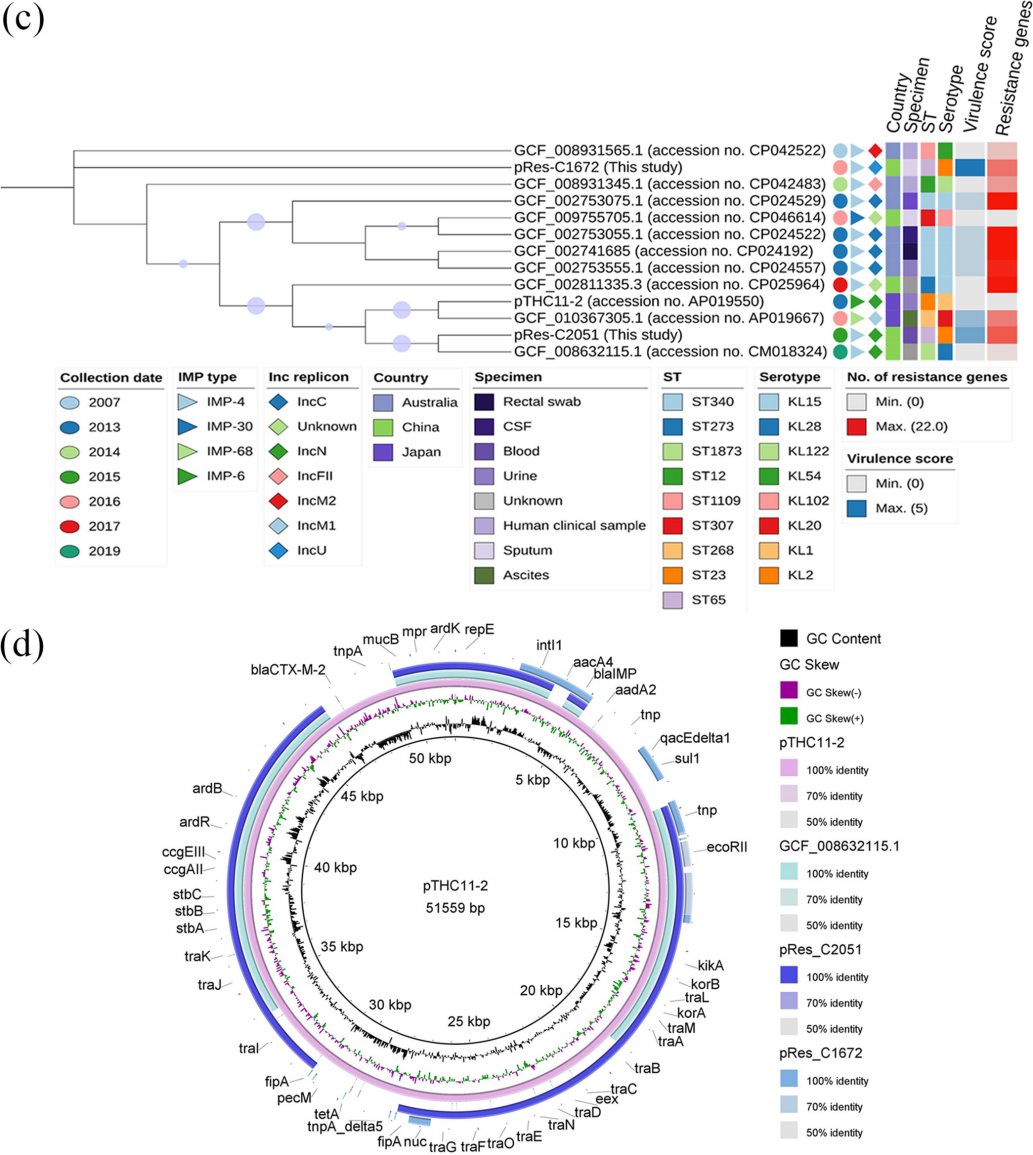
Comparative genomic analysis of the virulence and resistance plasmids. (a) Alignment analysis of virulence plasmid sequences among pVir-C1672, pVir-C2051, and previously reported virulence plasmids, including pLVPK (accession no. AY378100) and pTHC11-1 (accession no. AP019549). (b) The region containing resistance genes in pRes-C1672 and pRes-C2051. (c) Comparison of pRes-C1672 and pRes-C2051 resistance plasmids from this study with 10 complete resistance plasmids bearing *bla*_IMP_ that originated from K. pneumoniae strains and whose sequences were downloaded from the RefSeq database, as well as pTHC11-2 (accession no. AP019550). The maximum-likelihood phylogenetic tree was built by RaxML from an alignment generated by Roary and filtered to remove recombination using ClonalFrameML. The Interactive Tree Of Life (https://itol.embl.de) was used for visualization. The bootstraps are shown as blue circles on the branches. (d) Alignment analysis of resistance plasmid sequences among pRes-C1672, pRes-C2051, and previously reported resistance plasmids pTHC11-2 (accession no. AP019550) and GCF_008632115.1 (accession no. CM018324).

The whole-genome sequences of C1672 and C2051 were obtained by hybrid *de novo* assembly of short- and long-read sequencing data. The complete genome of strain C1672 was assembled into three contigs, including one chromosome (5,345,742 bp), one virulence plasmid (240,271 bp, designated pVir-C1672), and one multidrug resistance plasmid (328,921 bp, designated pRes-C1672) harboring *bla*_IMP-4_. Several resistance genes that mediate resistance to β-lactams (*bla*_SHV-12_), sulfonamides (*sul1* and *sul2*), aminoglycosides [*aac(6′)-Ib-cr*, *aac(3)-IId*, *aph(3′')-Ib*, *aph(6)-Id*, and *armA*], rifampin (*arr3*), quinolone (*qnrS1*), macrolides [*msr(E)* and *mph(E)*], and chloramphenicol (*catA2*) were also found in the resistance plasmid pRes-C1672. The genome assembly for C2051 was composed of a 5,280,186-bp chromosome and three plasmids, including one 236,472-bp virulence plasmid (designated pVir-C2051) and two resistance plasmids, one of which was a 54,214-bp resistance plasmid (designated pRes-C2051) bearing *bla*_IMP-4_ and *qnrS1* and the other one a 131,632-bp IncFII plasmid carrying *bla*_CTX-M-15_, *bla*_TEM-1_, *sul1*, *sul2*, *aph(3′')-Ib*, *aph(6)-Id*, *aph(3′)-Ia*, *aac(3)-IId*, *aac(6′)-Ib-cr*, *mph(A)*, *qnrB*, *aadA16*, *dfrA27*, and *arr-3*. Each isolate harbored a pLVPK-like virulence plasmid belonging to the IncHI1B/IncFIB replicon that was highly similar to plasmid pLVPK (GenBank accession no. AY378100), with more than 90% coverage and 99% identity (Fig. 3a). Another pLVPK-like plasmid, pTHC11-1 (accession no. AP019549), which was found in an IMP-6-producing K1-ST23 CR-hvKP isolate, was also used in the comparative analysis, and the results showed they all shared high similarity (Fig. 3a).

The *bla*_IMP_-bearing plasmids, pRes-C1672 and pRes-C2051, were not identical and belonged to the IncU and IncN replicons, respectively. The IncN replicon of C2051 was a canonical pIMP-4-like resistance plasmid that was highly similar to the plasmid bearing *bla*_IMP-4_ p13SP-IMP (GenBank accession no. MH909334.1), with more than 100% coverage and 99.95% identity, whereas *bla*_IMP-4_ was borne by a new ST65 IncU plasmid in the C1672 isolate. The *bla*_IMP-4_ gene was in a class 1 integron with the gene arrangement of the retron-type RNA-directed DNA polymerase-*bla*_IMP-4_-IntI1 (Fig. 3b). The genetic environments of the *bla*_IMP-4_ gene in pRes-C1672 and pRes-C2051 were very similar, except that the IS*6* family transposase was inserted in the downstream region of the *bla*_IMP-4_ gene in pRes-C2051 (Fig. 3b). Thus, we inferred that the genetic regions of *bla*_IMP-4_ were inserted into different backbones.

Since the conventional hypervirulent K. pneumoniae lineage (K2-ST65) has been associated with the carriage of a pLVPK-like plasmid, we speculated that C1672 and C2051 originally carried the virulence plasmids (pVir-C1672 and pVir-C2051) and acquired the resistance plasmids. We analyzed the phylogenetic information of the resistance plasmids (pRes-C1672 and pRes-C2051) from this study, 10 complete resistance plasmids bearing *bla*_IMP_ originating from K. pneumoniae whose sequences we downloaded from the RefSeq database, and pTHC11-2 (accession no. AP019550), which is an IncN plasmid harboring *bla*_IMP-6_ isolated from a K1-ST23 CR-hvKP strain in Japan, as mentioned above. The maximum-likelihood phylogenetic tree showed a link between pRes-C2051 and GCF_008632115.1 (accession no. CM018324), which was isolated from a K28-ST1873 carbapenem-resistant K. pneumoniae isolate from Hangzhou, China (Fig. 3c). To explain why pRes-C2051 could be transferred into the classic hypervirulent K. pneumoniae K2-ST65 lineage, we further compared pRes-C2051 with GCF_008632115.1 (accession no. CM018324) and pTHC11-2 (accession no. AP019550). Compared with GCF_008632115.1, several conjugal transfer genes, such as the *traO*, *traE*, *traN*, *traB*, *traC*, and *traD* genes, were identified in pRes-C2051, suggesting a higher ability for bacterial conjugation and transmission in C2051 (Fig. 3d).

In conclusion, we report the emergence of two IMP-4 carbapenemase-producing K2-ST65 hypervirulent K. pneumoniae isolates that caused severe and fatal infections in clinical settings in China. Notably, one of them also demonstrated tigecycline nonsusceptibility, most likely mediated by a frameshift mutation in the TetR/AcrR family transcriptional regulator. These strains, carrying *bla*_IMP_ and virulence plasmids, have the potential to exist for a long time without compromising their fitness, highlighting the urgent need to enhance clinical awareness and epidemiologic surveillance to generate essential data for preventing their dissemination.

## MATERIALS AND METHODS

### Bacterial isolates and detection of resistance and virulence genes.

A total of 3,285 carbapenem-resistant K. pneumoniae isolates were retrospectively collected from 26 provinces across China between 2014 and 2019. Species identification was confirmed by matrix-assisted laser desorption ionization–time of flight mass spectrometry (MALDI-TOF MS) (Bruker Daltonics, Billerica, MA, USA), and isolates were stored at −80°C until use. Carbapenem resistance was defined as nonsusceptibility to at least one carbapenem agent according to the CLSI breakpoints (imipenem, ≥2 mg/liter; meropenem, ≥2 mg/liter; and ertapenem, ≥1 mg/liter) or strains with carbapenemase production (20, 21).

All isolates were screened for the cocarriage of both a *bla*_IMP_-bearing resistance plasmid and a pLVPK-like virulence plasmid by detecting the carbapenemase gene *bla*_IMP_ and critical plasmid-borne virulence genes, including genes associated with hypermucoviscosity (*rmpA* and *rmpA2*), aerobactin (*iucA*), and salmochelin (*iroN*), using PCR and Sanger sequencing (3, 22). Positive results for all four of these virulence genes in a strain were considered an indicator of the full-length virulence plasmid according to previous studies (2). Two strains (C1672 and C2051) were enrolled in this study because they carried *bla*_IMP_ and all four virulence genes. Clinical information on cases involving these two strains was reviewed and collected, including demographics, underlying diseases, specimen types, clinical manifestations, antibiotic exposure histories, use of invasive devices, antimicrobial treatments, and outcomes.

### Antimicrobial susceptibility testing and microbiological features.

C1672 and C2051 were tested for antimicrobial susceptibility using the agar and broth microdilution method according to CLSI guidelines (23). Pseudomonas aeruginosa strain ATCC 27853 and Escherichia coli strain ATCC 25922 were used as controls. Data were interpreted using CLSI breakpoints, while the criteria for tigecycline followed those established by the U.S. Food and Drug Administration (21, 24). Multilocus sequence typing (MLST) was performed according to the protocol on the Pasteur Institute MLST website (https://bigsdb.pasteur.fr/klebsiella/klebsiella.html). K serotypes (K1, K2, K5, K20, K54, K57, K47, and K64) and hypermucoviscosity were also investigated as previously described (3).

### Serum killing assay.

A serum killing assay was conducted to determine *in vitro* virulence according to previous studies (25). An exponential-phase culture was diluted to 10^6^ CFU/ml. An inoculum of 50 μl of bacterial culture was added to 150 μl of pooled human sera obtained from 10 healthy individuals. Viable counts were checked at 0, 60, 120, and 180 min of incubation at 37°C and 200 rpm. The results were analyzed by comparing the CFU from each time point and expressed as either sensitive, intermediate, or resistant. Carbapenem-resistant K. pneumoniae strains from our previous study (3), C639 and C789, which were sensitive and resistant to serum killing, respectively, were used as negative and positive controls. Each strain was tested three times.

### Galleria mellonella infection model.

To determine the *in vivo* virulence, 250 to 350 mg of pathogen-free G. mellonella larvae were obtained from the Huiyude Biotech Company (Tianjin, China). A mid-log-phase bacterial culture was diluted using phosphate-buffered saline (PBS), and 10 μl (with a concentration of 10^7^ CFU/ml) was injected into the left proleg of each larvae using a microsample syringe (26). PBS, ST11 carbapenem-resistant K. pneumoniae strain ZR2 with low virulence, and K1-ST23 hypervirulent K. pneumoniae strain NTUH-K2044 were used as the blank, negative, and positive controls, respectively. After injection, larvae were kept at 37°C in the dark and mortality rates were observed for 72 h. Ten larvae were used as a sample population, and each strain was tested twice (27).

### Efflux pump inhibitory test.

The PAβN inhibitory test was used to explore whether tigecycline nonsusceptibility was mediated by efflux pumps (16, 28). The MIC of tigecycline (Pfizer, NY, USA) was determined according to CLSI recommendations using the broth microdilution method. A stock solution of PAβN (Sigma-Aldrich, Shanghai, China) was prepared at a concentration of 5 mg/ml in sterile water, with its final concentration in Mueller-Hinton broth at 25 mg/liter. Bacterial growth in Mueller-Hinton broth containing tigecycline with and without PAβN was detected in parallel. A growth control with 25 mg/liter PAβN in Mueller-Hinton broth was also added to check the effect of PAβN alone on each strain.

### Conjugation experiment.

To explore the presence of a plasmid-mediated resistance mechanism for tigecycline nonsusceptibility, conjugation experiments were performed using C2051 as the donor and E. coli strain J53 as the recipient, according to previous studies (18). The donor and recipient strains were mixed at a ratio of 1:1 for 24 h. The transconjugants were then selected on China blue lactose agar supplemented with tigecycline (1 mg/liter) and sodium azide (100 mg/liter). The transconjugants were confirmed by MALDI-TOF MS, and antimicrobial susceptibility was determined using the broth microdilution method.

### Stability assay of carbapenem resistance plasmid and fitness cost analysis.

Resistance plasmid stability was assessed as previously described (29). Briefly, the isolates were cultured in LB broth at 37°C with shaking (200 rpm), and then serial passaging was performed for 5 days with 1:1,000 dilutions in antibiotic-free LB broth. After 5 days, cultures were serially diluted and plated on MH agar plates without antibiotics or with ertapenem (2 mg/liter). The retention rate of the IMP-bearing plasmid was expressed as the percentage of CFU on the MH plate containing ertapenem compared to the number on the antibiotic-free plate. Colonies were selected and subjected to PCR for validation of *bla*_IMP-4_ genes.

Furthermore, to determine the potential effects of resistance plasmids on fitness, the growth kinetics of C1672/C2051 and their corresponding plasmid-cured mutants were investigated (18). Overnight cultures were diluted to an optical density at 600 nm (OD_600_) of ∼0.1 and grown at 37°C with 200-rpm shaking. The culture cell density was determined every 30 min by measuring the OD_600_.

### Whole-genome sequencing and bioinformatics analysis.

Genomic DNA of C1672 and C2051 was prepared using the TIANamp bacterial DNA kit (Tiangen Biotech, Beijing, China) and sequenced using the Illumina HiSeq platform and PacBio RS II system (Pacific Biosciences) to characterize the plasmid. The Unicycler pipeline version 0.4.7 was used for *de novo* assembly. The draft genome sequence was annotated using Rapid Annotation using Subsystem Technology (RAST) (http://rast.nmpdr.org/) and Prokka. Antimicrobial resistance genes, plasmid replicon types, virulence genes, serotypes, virulence scores, and insertion sequence (IS) elements were analyzed by using the Center for Genomic Epidemiology service (http://www.genomicepidemiology.org/) and several databases, including SerotypeFinder, PlasmidFinder, ResFinder, CARD, VirulenceFinder, VFDB, Kleborate (https://github.com/katholt/Kleborate), ICEberg, and ISFinder. BLAST Ring Image Generator (BRIG) and Easyfig tools were used to visualize the plasmid and genetic context comparisons.

### Phylogenetic analysis.

We compared the sequences of resistance plasmids bearing *bla*_IMP_ from our study with those of other complete resistance plasmids harboring *bla*_IMP_ isolated from K. pneumoniae strains (Table S1 in the supplemental material) that were downloaded from the RefSeq database. The plasmid sequences were aligned using Roary software (version 3.11.2) (30), and the recombinant regions of the alignments were removed using ClonalFrameML software (version 1.2) before phylogenetic analysis (31). Maximum-likelihood phylogenetic trees were constructed using RAxML software (version 8.2.10) and visualized using the Interactive Tree Of Life (https://itol.embl.de) (32, 33).

### Statistical analysis.

Statistical analysis was performed with one-way analysis of variance (ANOVA) using GraphPad Prism version 5. A *P* value of <0.05 was considered to be statistically significant.

### Ethics.

This study was approved by the Ethics Committee of Peking University People’s Hospital (reference number 2019PHE001).

### Data availability.

The complete sequences of the two strains in this study, C1672 and C2051, were deposited in the GenBank database (accession no. CP073917 to CP073919 and CP073920 to CP073923).
