# Supplementary material for: Emergence of Tigecycline Nonsusceptible and IMP-4 Carbapenemase-Producing K2-ST65 Hypervirulent Klebsiella pneumoniae in China
Source: Microbiol Spectr. 2021 Oct 27;9(2):e01305-21. doi: 10.1128/Spectrum.01305-21 (PMC8549734; doi:10.1128/Spectrum.01305-21)
Supplement: SUPPLEMENTAL FILE 1 — Supplemental material. Download SPECTRUM01305-21_Supp_1_seq2.pdf, PDF file, 0.03 MB [file spectrum01305-21_supp_1_seq2.pdf]

Supplementary tables

Table 1. Sequences of *bla*$_{IMP}$-encoding plasmids used for phylogenetic analysis in this study

assembly_ accession	accession No.	ST	Country	Collection date	Sample type	Inc replicon	virulence_ score*	K_locus	resistance_ score*	num_resistance_ genes	Bla_Carb
GCF_002741685.1	CP024192	ST340	Australia: Melbourne	2013/7/1	Rectal swab	IncC	1	KL15	2	22	IMP-4
GCF_002753055.1	CP024522	ST340	Australia: Melbourne	2013/8/26	CSF	IncC	1	KL15	2	22	IMP-4
GCF_002753075.1	CP024529	ST340	Australia: Melbourne	2013/8/26	Blood	IncC	1	KL15	2	22	IMP-4
GCF_002753555.1	CP024557	ST340	Australia: Melbourne	2013/9/3	Urine	IncC	1	KL15	2	21	IMP-4
GCF_002811335.3	CP025964	ST273	China: Sichuan, Chengdu	2017/7/1	Unknown	Unknown	0	KL15	2	22	IMP-4
GCF_008632115.1	CM018324	ST1873	China: Hangzhou	2019/3/9	Unknown	IncN	0	KL28	2	7	IMP-4
GCF_008931345.1	CP042483	ST12	Australia: Sydney	2014/5/30	human clinical sample	IncFII	0	KL122	2	12	IMP-4
GCF_008931565.1	CP042522	ST1109	Australia: Sydney	2007/1/17	human clinical sample	IncM2	0	KL54	2	9	IMP-4
GCF_009755705.1	CP046614	ST307	China	2016	Sputum	Unknown	0	KL102	2	6	IMP-30
GCF_010367305.1	AP019667	ST268	Japano	2016/7/12	Ascites	IncM1	2	KL20	2	14	IMP-68

assembly_ accession	accession No.	ST	Country	Collection date	Sample type	Inc replicon	virulence_ score*	K_locus	resistance_ score*	num_resistance_ genes	Bla_Carb
pTHC11-2	AP019550	ST23	Japan	2013	Urine	IncN	NA	K1	NA	NA	IMP-6
pRes-C1672	This study	ST65	China	2016/4/17	Sputum	IncU	5	K2	2	15	IMP-4
pRes-C2051	This study	ST65	China	2015/12/23	Blood	IncN	3	K2	2	17	IMP-4

Note:* The virulence and resistance scores were predicted by Kleborate software (https://github.com/katholt/Kleborate).
